# Day 3 Oxford criteria predict steroid non-response for acute severe ulcerative colitis in the post biologic era

**DOI:** 10.1093/ecco-jcc/jjaf131

**Published:** 2025-07-18

**Authors:** Sudheer K Vuyyuru, Lotus Alphonsus, Theshani Amalka De Silva, Virginia Solitano, Leonardo Guizzetti, Terry Ponich, Melanie Beaton, Jamie Gregor, Brian Yan, Michael Sey, Vipul Jairath

**Affiliations:** Division of Gastroenterology, Department of Medicine, Western University, London, Ontario, Canada; Schulich School of Medicine and Dentistry, Western University, London, Ontario, Canada; Schulich School of Medicine and Dentistry, Western University, London, Ontario, Canada; Division of Gastroenterology, Department of Medicine, Western University, London, Ontario, Canada; Division of Gastroenterology and Gastrointestinal Endoscopy, IRCCS Ospedale San Raffaele, Milan, Italy, Università Vita-Salute San Raffaele, Milan, Italy; Independent Researcher, London, Ontario, Canada; Division of Gastroenterology, Department of Medicine, Western University, London, Ontario, Canada; Division of Gastroenterology, Department of Medicine, Western University, London, Ontario, Canada; Division of Gastroenterology, Department of Medicine, Western University, London, Ontario, Canada; Division of Gastroenterology, Department of Medicine, Western University, London, Ontario, Canada; Division of Gastroenterology, Department of Medicine, Western University, London, Ontario, Canada; Lawson Health Research Institute, Western University, London, Ontario, Canada; Division of Gastroenterology, Department of Medicine, Western University, London, Ontario, Canada; Lawson Health Research Institute, Western University, London, Ontario, Canada; Department of Epidemiology and Biostatistics, Western University, London, Ontario, Canada

**Keywords:** ulcerative colitis, infliximab, cyclosporine, colectomy

## Abstract

**Background and aims:**

Outcomes of patients admitted with acute severe ulcerative colitis (ASUC) in the post biologic era are under explored, as well as the ability of scoring indices to predict early steroid non-response.

**Methods:**

This retrospective cohort study included adults hospitalized with ASUC (2010-2022) at London Health Sciences Centre, Canada. Steroid response, need for rescue therapy, colectomy during index hospitalization, and colectomy and hospitalization at 3- and 12-months following discharge was assessed. Logistic regression identified predictors of steroid non-response, defined as need for rescue therapy or colectomy during hospitalization.

**Results:**

Of 261 adults hospitalized with ASUC (male: 51.7%, mean age: 40.6 years), 71.2% had extensive colitis. After intravenous corticosteroid therapy during index admission, 55.7% (*n *= 147) had a response, 37.9% (*n *= 99) received rescue therapy (infliximab: 98, tofacitinib: 1, and cyclosporine: 0), and 8% (21/261) underwent colectomy. Additionally, 11.6% (28/240) of patients discharged from hospital underwent colectomy within the first 12 months (8.3% at 3-months and 3.3% between 3 and 12 months). There was no difference between steroid responders and non-responders for colectomy (11% vs 12.6%) or hospitalization (33.5% vs 32.6%) at 12 months. The overall cumulative probabilities of colectomy for the entire cohort at 1 year, 3 years, and 5 years were 13.5%, 16.1%, and 17.4%, respectively. On multivariate analysis, Day 3 Oxford criteria was the only factor found to be statistically significant in predicting steroid non-response (odds ratio 4.70, 95%CI [1.06-20.80]).

**Conclusions:**

Day 3 Oxford criteria was an independent predictor of steroid non-response. The risk of colectomy remains substantial after discharge despite low in-hospital colectomy rates following an episode of ASUC. Initial steroid response did not affect long-term colectomy rate at 12 months.

## 1. Introduction

Acute severe ulcerative colitis (ASUC) is a severe exacerbation of ulcerative colitis (UC) disease activity, characterized by clinical symptoms of bloody diarrhoea and abdominal discomfort accompanied by features of systemic inflammation, such as elevated C-reactive protein (CRP) or erythrocyte sedimentation rate (ESR) levels.^1^^,^^2^ ASUC is a potentially life-threatening condition and can result in serious complications such as toxic megacolon, and colonic perforation if not treated expediently. Since the introduction of intravenous (IV) corticosteroids in the management algorithm over 70 years ago,^2^ it remained the cornerstone first line therapy. However, only up to two thirds of patients respond to IV corticosteroids necessitating additional medical rescue therapy.^3^ In medically refractory cases, emergency subtotal colectomy is required as a surgical rescue therapy.

ASUC is reported to affect 25% of patients with UC during their lifetime and 30% of these are first presentations of UC.^4^ Up to 10%-15% of patients would require emergency colectomy as a surgical rescue therapy during an episode of ASUC.^5^^,^^6^ The risk of colectomy increases with subsequent episodes of ASUC.^4^ The goal of ASUC management is rapid induction of a response with medical therapy in order to avoid emergency colectomy which can be associated with poor outcomes compared to colectomy in an elective setting. Accordingly, early identification of patients who may not respond to IV corticosteroids is crucial so that these patients receive more intensive management and intervention, rather than waiting for the standard 3-5 days of corticosteroids to assess response as is recommended in guidelines.^7^^,^^8^

There are several indices that have been developed to predict outcomes in patients with ASUC, which include predicting steroid non-response, response to medical rescue therapy, and to predict outcomes after discharge.^9^ However, none of these indices are fully validated. Among these, the Oxford criteria were developed in 1996 by Travis and colleagues to predict the risk of colectomy on the same admission, based on stool frequency (SF) and CRP.^10^ Although it was developed to predict colectomy, it is often used to predict response to IV corticosteroids on Day 3 in order to inform the need for rescue therapy.

In the post biologic era, the management of UC has evolved since the introduction of advanced medical therapies. Currently there are 7 monoclonal antibodies (infliximab, adalimumab, golimumab, vedolizumab, ustekinumab, risankizumab, and mirikizumab) and 5 oral small molecules (filgotinib, tofacitinib, upadacitinib ozanimod, and etrasimod) that are approved for the management of moderate to severely active UC across various jurisdictions.^11^ Consequently, it is not uncommon to manage patients admitted with ASUC who have failed prior advanced therapies. A recent meta-analysis of population-based cohort studies indicated that the cumulative risk of colectomy has reduced over the years possibly because of widespread availability of advanced therapies.^12^ However, the impact of these therapies on colectomy following an episode of ASUC is not clear. Therefore, in this study we aimed to assess short- and long-term outcomes in patients admitted with ASUC in the post biologic era as well as to evaluate the performance of predictive indices for steroid non-response.

## 2. Methods

### 2.1. Study design

This was a retrospective, observational study conducted at London Health Sciences Center, London, Ontario, Canada, analyzing data from patients hospitalized with ASUC between 2010 and 2022. The study was designed with two main goals: (1) to describe colectomy rates during the same admission as well as up to 12 months post-discharge after ASUC in the post biologic era; (2) to evaluate the performance of established scoring indices in predicting non-response to IV corticosteroids.

The inclusion criteria were: (i) adults aged ≥18 years hospitalized with ASUC; (ii) receiving at least 3 days of IV corticosteroid therapy; (iii) hospitalized for at least 3 days. The diagnosis of ASUC and decision to admit was determined by the treating physician based on clinical, laboratory, and endoscopic findings.

### 2.2. Data collection

All data were recorded using standardized case report forms, de-identified, and entered into a central database registry: the Research Electronic Data Capture (REDCap) database. Data were extracted from electronic hospital records as well as from physical chart reviews, including demographic information, clinical parameters, laboratory results, and treatment outcomes including need for rescue therapy and need for colectomy. Patients were classified as steroid responders or steroid non-responders based on the requirement for medical rescue therapy or colectomy.

### 2.3. Statistical analysis

Descriptive statistics were used to summarize the data. Continuous variables were expressed as means and medians. Comparative analyses between steroid responders and non-responders were conducted using appropriate statistical tests. Univariate and multivariate logistic regression analyses were performed to identify predictors of steroid non-response. A backward selection procedure was employed, wherein variables were retained in the model only if their *P*-value remained <.15. CRP and SF were analyzed as grouped variables to jointly test their Day 1 values and changes from Day 1 to Day 3. This grouping approach accounted for the possibility that only the changes over time, rather than the baseline values, might demonstrate predictive significance. By applying this method, we ensured a comprehensive assessment of both baseline and dynamic factors influencing non-response to IV steroids. Changes in CRP and SF were evaluated as potential predictors of steroid response. The Liu method was applied to determine optimal cutoff values for these variables.^13^ The sensitivity and specificity of the Oxford criteria, CRP, and albumin in predicting steroid non-response were calculated to assess their diagnostic performance.

### 2.4. Handling of missing data

A sensitivity analysis was conducted using multiple imputation by chained equations using 50 completed datasets. Numerical instabilities were encountered during some of these multiple imputation analyses. As a result, imputation considered the following reduced set of variables: Day 1 CRP, Day 1 albumin, Day 1 Lindgren score, change from Day 1 to Day 3 CRP, change from Day 1 to Day 3 stool frequency, Day 3 steroid non-response per the Oxford criteria, thromboprophylaxis use, presenting within 1 year of onset, age, sex, and prior biologic use. (Variations of these models considered using Day 1 and Day 3 CRP, or Day 1 and Day 3 stool frequency, but these variables were too collinear, and the imputation model would not converge.) The variations of these imputation models yielded similar results.

### 2.5. Ethical considerations

This study was approved by the institutional review board. Patient confidentiality was maintained by anonymizing all data in compliance with ethical guidelines.

## 3. Results

### 3.1. Baseline characteristics at presentation

A total of 261 adults hospitalized with ASUC were included in the analysis (Table 1). The mean age of the cohort at the time of admission was 40.6 ± 17.6 years and 51.7% (*n *= 135) were male. The majority of patients had extensive colitis (71.2% [*n *= 168]) at the time of diagnosis. The mean disease duration prior to admission was 5.6 ± 8.2 years and 45.8% (*n*/*N *= 116/253) presented within 1 year of symptom onset. ASUC was the first presentation of UC for 23% (*n *= 61) of patients. One fifth of patients (23.3% [*n*/*N *= 61/261]) had experienced advanced therapies and 25.9% (*n*/*N *= 66/255) had experienced immunomodulators prior to ASUC presentation. In total 20% (*n*/*N *= 51/253) of the cohort were steroid dependent upon admission.

**Table 1.** Demographics and baseline characteristics.

Parameter	Overall (*N *= 261)	Steroid responders	Steroid non-responders	*P*-value
		(*n *= 145)	(*n *= 116)	
**Age at admission (years) Mean (SD)**	40.6 (17.6)	42.9 (18.6)	37.7 (15.9)	.0158
**Sex, *n* (%)**				
** Male**	135 (51.7)	75 (51.7)	60 (51.7)	1.0000
** Female**	126 (48.3)	70 (48.3)	56 (48.3)	
**Smoking status, *n*/*N* (%)**				
** Never**	159 (76.4)	82 (72.6)	77 (81.1)	.1832
** Ex-smoker**	36 (17.3)	21 (18.6)	15 (15.8)	
** Current**	13 (6.2)	10 (8.8)	3 (3.2)	
**Extent, *n*/*N* (%)**				
** Left sided colitis**	68 (28.8)	37 (27.6)	31 (30.1)	.7268
** Extensive colitis**	168 (71.2)	97 (72.4)	71 (69.6)	
**Disease duration (years) (*n*/*N*)**	253	140	113	
** Mean (SD)**	5.6 (8.2)	6.3 (9.4)	4.7 (6.4)	.0929
**Presented within first year of onset, *n*/*N* (%)**	116/253 (45.8)	58/140 (41.4)	58/113 (51.3)	.1162
**Prior therapies, *n*/*N* (%)**				
** Immunosuppressants**	66/255 (25.9)	31/139 (22.3)	35/116 (30.2)	.1530
** Infliximab**	46/254 (18.1)	29/140 (20.7)	17/114 (14.9)	.2324
** Adalimumab**	14/254 (5.5)	7/140 (5.0)	7/114 (6.1)	.6920
** Vedolizumab**	25/254 (9.8)	16/140 (11.4)	9/114 (7.9)	.3470
** Ustekinumab**	1/254 (0.4)	0/140 (0.0)	1/114 (0.9)	.2668
** Tofacitinib**	5/251 (2.0)	4/137 (2.9)	1/114 (0.9)	.2489
**Steroid dependent** ^a^ **, *n*/*N* (%)**	51/253 (20.2)	25/139 (18.0)	26/114 (22.8)	.3415
**Abdominal pain, *n*/*N* (%)**				.4216
** None**	36/227 (15.9)	22/124 (17.7)	14/103 (13.6)	
** Mild**	78/227 (34.4)	44/124 (35.5)	34/103 (33.0)	
** Moderate**	86/227 (37.9)	47/124 (37.9)	39/103 (37.9)	
** Severe**	27/227 (11.9)	11/124 (8.9)	16/103 (15.5)	
**Enteric infections on stool culture, *n*/*N* (%)**	3/204 (1.5)	0/113 (0.0)	3/91 (3.3)	.0518
***C.difficile* infection, *n*/*N* (%)**	11/213 (5.2)	7/118 (5.9)	4/95 (4.2)	.5725
**CMV infection (based on inclusion bodies on biopsy), *n*/*N* (%)**	8/134 (6.0)	4/61 (6.6)	4/73 (5.5)	.7931
**CT findings, *n*/*N* (%)**				.6878
** Normal**	3/85 (3.5)	2/39 (5.1)	1/46 (2.2)	
** Transmural thickening**	74/85 (87.1)	34/39 (87.2)	40/46 (87.0)	
** Distended colon**	8/85 (9.4)	3/39 (7.7)	5/46 (10.9)	
**Parenteral nutrition, *n*/*N* (%)**	35/252 (13.9)	7/139 (5.0)	28/113 (24.8)	.0000
**Nutritional supplements *n*/*N* (%)**	25/261 (9.6)	7/145 (4.8)	18/116 (15.5)	.0035
**Thromboprophylaxis, *n*/*N* (%)**	168/250 (67.2)	82/137 (59.9)	86/113 (76.1)	.0064
**Formal surgical consultation, *n*/*N* (%)**	73/244 (29.9)	18/133 (13.5)	55/111 (49.5)	.0000

a Steroid dependence—prolonged usage of steroids for >3 months or need for reinitiation within 3 months of stopping.

Abbreviations: CMV, cytomegalovirus; CT, computerized tomography; SD, standard deviation.

### 3.2. Disease activity on Day 1

On Day 1 of hospitalization, the mean SF was 11.9 ± 6.0 per day and the majority of patients (91.7% [*n*/*N *= 233/254]) had bloody stools. The majority (79.3% [*n*/*N *= 207/261]) satisfied the Truelove and Witts’ criteria for ASUC, of whom 49.2% (*n*/*N *= 197/258) had 2 or more systemic inflammatory features. In the remaining 54 patients, Truelove and Witts’ criteria could not be determined due to absence of data on number of bloody stools. However, 43 patients had at least 1 systemic criterion for severe UC. Mean serum albumin level of the cohort was 31.2 ± 6.0 g/L and median CRP was 61.5 mg/L (inter quartile range [IQR]: 20.9-122.7). Median CRP/albumin ratio was 2.0 (0.8-3.9), median SEO index was 224.6 (IQR: 200.4-244.8), and median Lindgren index was 21.9 (IQR: 14.3-30.6) on Day 1 (Table 2).

**Table 2.** Disease activity on day 1.

Parameter	Overall (*N *= 261)	Steroid responders	Steroid non-responders	*P*-value
		(*n *= 145)	(*n *= 116)	
**Stool frequency, *n***	255	140	115	
** Mean (SD)**	11.9 (6.0)	11.7 (5.8)	12.0 (6.2)	.6884
** Median (IQR)**	10.0 (7.0-15.0)	10.0 (7.0-15.0)	10.0 (7.0-15.0)	
**Blood in stools, *n*/*N* (%)**	233/254 (91.7)	127/140 (90.7)	106/114 (93.0)	.5138
**Truelove and Witts’ criteria, *n*/*N* (%)**				
** Tachycardia (>90 bpm)**	144/249 (57.8)	75/134 (56.0)	69/115 (60.0)	.5209
** CRP >30 mg/L**	140/204 (68.6)	75/111 (67.6)	65/93 (69.9)	.7215
** Fever (>37.8 °C)**	20/245 (8.2)	11/133 (8.3)	9/112 (8.0)	.7215
** Hemoglobin <105 g/L**	73/242 (30.2)	40/132 (30.3)	33/110 (30.0)	.9592
**Number of positive TW criteria**				
** 0**	48/258 (18.6)	27/142 (19.0)	21/116 (18.1)	.4033
** 1**	83/258 (32.2)	48/142 (33.8)	35/116 (30.2)	
** 2**	88/258 (34.1)	49/142 (34.5)	39/116 (33.6)	
** 3**	38/258 (14.7)	17/142 (12.0)	21/116 (18.1)	
** 4**	1/258 (0.4)	1/142 (0.7)	0/116 (0.0)	
**Albumin (g/L), *n***	168	92	76	
** Mean (SD)**	31.2 (6.0)	31.2 (6.4)	31.1 (5.5)	.9164
** Median (IQR)**	30.0 (27.0-36.0)	30.0 (27.0-37.0)	30.5 (28.0-34.5)	
**Hemoglobin (g/L), *n***	242	132	110	
** Mean (SD)**	114.8 (22.8)	113.6 (23.0)	116.3 (22.7)	.3738
** Median (IQR)**	115.0 (101.0-131.0)	114.0 (101.0-131.5)	116.5 (101.0-130.0)	
**CRP (mg/L), *n***	204	111	93	
** Mean (SD)**	83.9 (77.4)	86.9 (85.9)	80.3 (66.2)	.5369
** Median (IQR)**	61.5 (20.9-122.7)	56.4 (16.2-136.9)	61.5 (22.5-119.9)	
**ESR (mm/hr), *n***	110	72	38	
** Mean (SD)**	39.0 (26.5)	38.9 (26.8)	39.4 (26.3)	.9203
** Median (IQR)**	33.5 (17.0-57.0)	33.0 (16.0-57.5)	33.5 (19.0-49.0)	
**CRP/Albumin ratio, *n***	145	80	65	
** Mean (SD)**	2.8 (2.8)	3.0 (3.2)	2.5 (2.1)	.2832
** Median (IQR)**	2.0 (0.8-3.9)	1.9 (0.6-4.4)	2.2 (0.8-3.9)	
**SEO index, *n***	78	50	28	
** Mean (SD)**	222.1 (28.6)	223.3 (29.9)	220.1 (26.7)	.6364
** Median (IQR)**	224.6 (200.4-244.8)	227.2 (200.4-245.0)	219.9 (203.5-239.7)	
**Lindgren index, *n***	200	108	92	
** Mean (SD)**	23.3 (11.9)	23.8 (13.2)	22.8 (10.3)	.5605
** Median (IQR)**	21.9 (14.3-30.6)	21.8 (12.9-31.4)	22.3 (15.1-29.2)	

Abbreviations: CRP, C-reactive protein; ESR, erythrocyte sedimentation rate; IQR, interquartile range; SD, standard deviation.

### 3.3. Outcomes during hospital stay

During hospital stay, 1.5% (*n*/*N *= 3/204) had positive enteric infection on stool culture and 5.2% (*n*/*N *= 11/213) were positive for *Clostridium difficile* toxin. Cytomegalovirus inclusion bodies on histopathological examination of colonic/rectal biopsies were observed in 6% (*n*/*N *= 8/134). Of 85 patients who underwent abdominal CT imaging during their hospital stay, transmural thickening was present in 87.1% (*n*/*N *= 74/85) and a distended colon was seen in 9.4% (8/85). Parenteral nutrition was administered to 13.9% (*n*/*N *= 35/252) of patients during hospitalization and two thirds (67.2% [*n*/*N *= 168/250]) received thromboprophylaxis. Early surgical consultation was obtained in 29.9% (*n*/*N *= 73/244). Endoscopic assessment of disease severity was performed in 77.8% (*n*/*N *= 203/261). Of these, 59.3% (155/261) had flexible sigmoidoscopy within the first 3 days of presentation and 76% (*n*/*N *= 108/142) had a Mayo endoscopic subscore of 3. On histopathological examination 38% (*n*/*N *= 46/119) had features of severe colitis. During hospital stay, 55.5% (*n *= 145) responded to IV corticosteroids and median hospital stay (IQR) was 7 days. Medical rescue therapy was needed in 39% (*n*/*N *= 101/258) of patients and 8% (*n*/*N *= 21/261) required colectomy during the index hospital admission. Infliximab was the most common rescue agent (97%, *n*/*N *= 98/101), and of these, 19 patients received either an intensified dose of infliximab (10 mg/kg) or accelerated dosing (2 doses of infliximab within 1 week). Only 1 patient received tofacitinib as rescue therapy and none received cyclosporine.

### 3.4. Predictors of steroid non-response

Various clinical, laboratory, endoscopic, and histological parameters were compared between steroid responders and non-responders. There were no differences in baseline characteristics between steroid responders and non-responders (Tables 1 and 2). However, on Day 3 of IV corticosteroid therapy, non-responders had higher median SF, higher proportion of blood in stools, higher median CRP, and lower median albumin levels compared to steroid responders. Similar trends were observed on Day 5 as well (Table 3).

**Table 3.** Disease activity on day 3.

Parameter	Overall	Steroid responders	Steroid non-responders	*P*-value
**Severity on sigmoidoscopy in first 3 days**				
** Mayo endoscopic score 1**	4/142 (2.8)	4/81 (4.9)	0/61 (0.0)	.1361
** Mayo endoscopic score 2**	30/142 (21.1)	19/81 (23.5)	11/61 (18.0)	
** Mayo endoscopic score 3**	108/142 (76.1)	58/81 (71.6)	50/61 (82.0)	
**Histopathological severity on biopsy in first 3 days**				
** Mild colitis**	16/119 (13.4)	10/67 (14.9)	6/52 (11.5)	.7157
** Moderate colitis**	57/119 (47.9)	30/67 (44.8)	27/52 (51.9)	
** Severe colitis**	46/119 (38.7)	27/67 (40.3)	19/52 (36.5)	
**Stool frequency, *n***	232	128	104	
** Mean (SD)**	6.6 (4.1)	5.4 (3.4)	8.1 (4.4)	.0000
** Median (IQR)**	6.0 (3.0-8.5)	4.0 (3.0-7.0)	7.0 (5.0-10.0)	
**Blood in stools, *n*/*N* (%)**	163/229 (71.2)	77/126 (61.1)	86/103 (83.5)	
**Albumin (g/L), *n***	75	39	36	
** Mean (SD)**	29.9 (10.2)	30.6 (12.2)	29.2 (7.6)	.0002
** Median (IQR)**	29.0 (25.0-32.0)	30.0 (24.0-33.0)	29.0 (25.0-31.0)	.5594
**Hemoglobin (g/L), *n***	248	135	113	
** Mean (SD)**	106.4 (19.9)	107.6 (20.8)	104.9 (18.7)	.2846
** Median (IQR)**	105.5 (91.0-120.0)	106.0 (91.0-122.0)	105.0 (89.0-119.0)	
**CRP (mg/L), *n***	137	73	64	
** Mean (SD)**	52.2 (53.6)	43.7 (46.3)	62.0 (59.7)	.0494
** Median (IQR)**	32.6 (15.7-75.2)	25.0 (9.9-56.4)	43.4 (19.1-86.9)	
**CRP/Albumin ratio, *n***	45	21	24	
** Mean (SD)**	1.8 (1.9)	1.5 (2.0)	2.1 (1.8)	.2872
** Median (IQR)**	1.3 (0.5-2.3)	0.8 (0.2-1.3)	1.6 (0.6-3.2)	
**Lindgren index, *n***	122	64	58	
** Mean (SD)**	13.7 (8.6)	11.4 (7.5)	16.3 (9.0)	.0015
** Median (IQR)**	11.6 (7.6-17.4)	9.4 (5.7-14.6)	14.4 (9.3-20.2)	
**Oxford criterion, *n*/*N* (%)**	52/122 (42.6)	18/64 (28.1)	34/58 (58.6)	.0007

Abbreviations: CRP, C-reactive protein; IQR, interquartile range; SD, standard deviation.

On univariate analysis, SF on Day 3, magnitude of change (Δ) in SF, and Δ CRP from Day 1 to Day 3, a Lindgren index of ≥8 on Day 3, thromboprophylaxis, total parenteral nutrition, satisfying Oxford criteria (SF >8, or 3-8 with CRP >45 mg/L) on Day 3 were predictive of steroid non-response. However, on multivariate analysis Oxford criteria was the only factor that remained statistically significant for predicting steroid non-response (Table 4). Among 80 patients who had data on Day 1 CRP, albumin, and endoscopic severity, 22 had an ACE index score of 3. Out of these 22 patients 8 (36.4%) did not respond to IV corticosteroids.

**Table 4.** Univariable and multivariable analysis of early predictors of steroid non-response.

Parameter	Univariable analysis			Multivariable model	
	*N*	OR (95% CI)	*P*-value	OR (95% CI)	*P*-value
**Index presentation as ASUC**	253	0.85 (0.47, 1.53)	.5930		
**ASUC with first year of symptom onset**	253	1.49 (0.91, 2.46)	.1168		
**Day 1 CRP, for each 10 mg/L increase**	204	0.99 (0.95, 1.03)	.5443	1.04 (0.91, 1.20)	.555
**Day 1 CRP >100 mg/L**	204	0.99 (0.55, 1.79)	.9788		
**Day1 stool frequency**	255	1.01 (0.97, 1.05)	.6850	0.97 (0.83, 1.13)	.7157
**Albumin <30 gm/L on Day 1**	168	0.64 (0.34, 1.18)	.1531		
**HB <105 on Day 1**	242	0.99 (0.57, 1.71)	.9592		
**Day1 SEO index**	78	1.00 (0.98, 1.01)	.6420		
**Day1 Lindgren index**	200	0.99 (0.97, 1.02)	.5662		
**CRP/Albumin ratio ≥0.85**	145	1.14 (0.55, 2.38)	.7280		
**Number of Truelove and Witts’ criteria on Day 1**	258	1.11 (0.86, 1.44)	.4018		
**Day 3 stool frequency**	232	1.21 (1.12, 1.31)	.0000		
**Change (Δ) in stool frequency from Day 1 to Day 3**	230	1.06 (1.01, 1.11)	.0089	0.96 (0.82, 1.12)	.5885
**Change (Δ) in CRP from Day 1 to Day 3, for each 10 mg/L increase**	109	1.08 (1.02, 1.15)	.0127	1.13 (0.96, 1.33)	.1289
**Day 3 CRP, for each 10 mg/L increase**	137	1.07 (1.00, 1.15)	.0523		
**Day3 Lindgren index**	122	1.08 (1.03, 1.14)	.0029		
**Fulfilling Oxford criteria on Day 3**	122	3.62 (1.70, 7.70)	.0008	4.70 (1.06, 20.80)	.0414
**Positive for *C. difficile***	213	0.70 (0.20, 2.46)	.5743		
**Total parenteral nutrition**	252	6.21 (2.60, 14.86)	.0000		
**Thromboprophylaxis**	250	2.14 (1.23, 3.71)	.0069	2.55 (0.98, 6.67)	.0562

Abbreviations: ASUC, acute severe ulcerative colitis; CRP, C-reactive protein; HB, hemoglobin.

Satisfying Oxford criteria on Day 3 (SF >8, or 3-8 with CRP >45 mg/L) had a sensitivity, specificity, and area under curve (AUC) of 58.6%, 71.9%, and 65.2%, respectively, for predicting steroid non-response during index hospital admission. Optimal cut offs for Δ SF and Δ CRP from Day 1 to Day 3 for predicting response to corticosteroids were also calculated. A decrease in SF by ≥3 had a sensitivity and specificity of 76.2% and 44.2%, respectively. A ≥ 12.2 mg/L decrease in CRP had sensitivity (64.3%) and specificity (54.7%) for predicting steroid response.

### 3.5. Short- and long-term outcomes following ASUC episode

**Figure 1.**
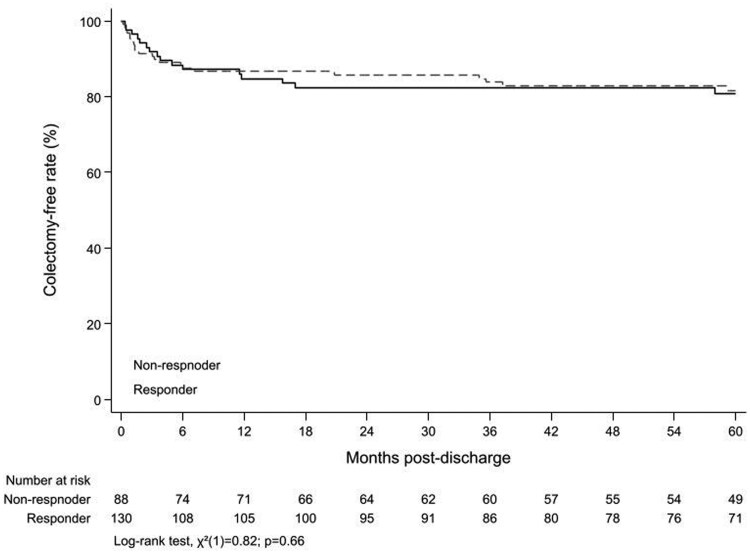
Kaplan–Meier estimates of colectomy-free rates, by steroid responder status.

**Figure 2.**
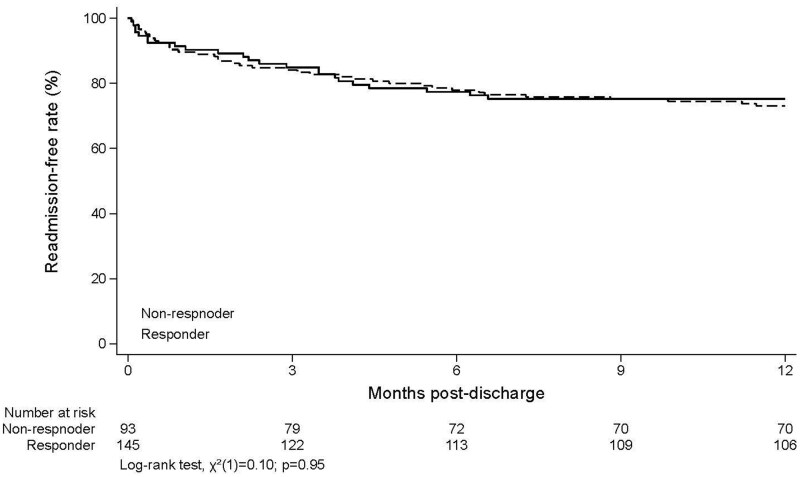
Kaplan–Meier estimates of readmission-free rates for exacerbations based on steroid responder status.

After excluding those patients who had undergone colectomy during index admission, the remaining patients (*n *= 240) were analyzed for short- (3 months) and long-term (12 months) outcomes. Overall, at 12 months following discharge, 32.9% (79/240) required hospital re-admission with exacerbation and 11.6% (28/240) of patients underwent colectomy. Of these patients who required colectomy, the majority underwent this within the first 3 months (8.3%) compared to 3-12 months (3.3%). There was no difference between steroid responders and steroid non-responders for colectomy (11% vs 12.6%) or hospitalization (33.5% vs 32.6%) at 12 months following discharge. However, notably all steroid non-responders (except those who underwent colectomy during index admission) continued to receive biologics (infliximab: 85, tofacitinib: 1, adalimumab: 1, vedolizumab: 1, switched from 1 biologic to another in 1 year: 4) whereas only 51% (*n*/*N *= 74/145) of steroid responders received biologics following discharge (infliximab: 36, adalimumab: 7, golimumab: 2, vedolizumab: 15, ustekinumab: 4, risankizumab: 1, and switched from 1 biologic to another in 1 year: 9). On survival analysis, there was no difference between steroid responders and non-responders in colectomy or hospitalization rates at 12 months (Figures 1 and 2). Moreover, the colectomy rate among steroid responders who received advanced therapies after discharge was similar (10.6%) to colectomy rate (11.4%) in those who did not receive advanced therapies at 12 months. The overall cumulative probabilities of colectomy of the entire cohort at 1 year, 3 years, and 5 years were 13.5%, 16.1%, and 17.4%, respectively. These probabilities were slightly higher in steroid non-responders compared to steroid responders but was not statistically significant (*P* = .66) (Figure 2).

### 3.6. Outcomes based on prior exposure to advanced therapy

Of the 261 patients included in the study, 61 (23.4%) had prior exposure to advanced therapies. The steroid response rates were 62.3% and 53.5% in patients with and without prior exposure to advanced therapies, respectively, whereas the colectomy rate among patients with prior exposure to advanced therapies was 18% during index hospitalization and 19.7% in the first 12 months post-discharge. In contrast, among patients naïve to advanced therapies, the colectomy rate was 5% during index hospitalization and 8% in the first 12 months following discharge.

## 4. Discussion

The present study informs important short- and long-term outcomes for patients hospitalized with ASUC in a real-world setting in the post biologic era. In this cohort, approximately one third of patients had prior exposure to advanced therapies and one quarter had experienced immunosuppressive medications at baseline. During index hospital admission, 55% responded to IV corticosteroids, 39% of patients required medical rescue therapy, and 8% underwent colectomy during the same hospital admission. Additionally, 11.6% (28/240) of patients discharged from hospital underwent colectomy within the first 12 months (8.3% at 3 months and 3.3% between 3 and 12 months). Overall colectomy and hospitalizations (33.5% vs 32.6%) at 12 months post discharge was similar between steroid responders and non-responders (11% vs 12.6%). The novel findings were that Oxford criteria was the only factor that remained statistically significant on ­multivariate analysis for predicting non-response to IV corticosteroids.

In the last few decades, there has been significant improvement in outcomes for ASUC reflected by a decrease in mortality from 28% in the1950s to 1% in the post biologic era.^14^^,^^15^ Similarly, there has been an improvement in colectomy rates at least in the short-term following an episode of ASUC, although response to IV corticosteroids has remained almost identical.^3^ In the Oxford cohort between 1992 and 1993, prior to the biologic era, the colectomy rate during hospital admission was 29% which had reduced to 21% in a UK-wide audit study and to approximately 15% in a more recent cohort (2015-2019).^16^ This is likely due to the availability of infliximab as a rescue option which has been shown to be effective in steroid refractory cases of ASUC.^17^^,^^18^ Whether this reduced short-term colectomy rate during index hospital admission has translated to a reduction in longer-term colectomy is debatable. In a UK-wide population-based study, a consistent reduction in short-term emergency colectomy rates was reported, but these reductions did not persist beyond 1 year.^19^

The risk of colectomy following discharge remains high following an episode of ASUC especially when patients did not adequately respond to IV corticosteroids during hospital stay. In the Oxford cohort from 1992-1993, patients who had complete response (SF ≤3/day on Day 7, with no visible blood) to IV corticosteroids had a 5% risk of colectomy compared to a 40% chance of colectomy for incomplete responders (SF >3 or visible blood on Day 7 who did not require colectomy on that admission).^10^ Similarly, in another retrospective cohort study, the probability of colectomy free survival was 96% at 1 year in those who responded to IV corticosteroids despite the relapse free survival rate being only 58%.^20^ Although the risk of colectomy had reduced to 17% at 1 year in patients who needed rescue therapy from the more recent Oxford cohort (2015-2019), it was still considerably high.^16^ Interestingly in our study the need for colectomy (11% vs 12.6%) and hospitalization (33.5% vs 32.6%) following ASUC was similar between steroid responders and non-responders. The most plausible explanation for this observation is due to the use of biological therapy in steroid non-responders. In our cohort almost all steroid non-responders continued to receive biologic therapy after discharge. This suggests a protective effect of biologics on post ASUC discharge colectomy rates.

Several indices have been developed to predict response to corticosteroids, need for rescue therapy, short- and long-term colectomy rates.^9^ The majority of these indices were developed from retrospective data and were not prospectively validated. The Oxford criteria was developed based on data from a prospective cohort and incorporated SF and CRP and is simple to use. As per the criteria, 85% of patients with SF of >8 or SF between 3-8 with CRP >45 mg/L on Day 3 would require colectomy. However, with increasing use of second line rescue therapy the in-hospital colectomy rates have reduced. For instance, in a UK-wide IBD audit data (2010-2011), in which only 34% of patients satisfying the Oxford criteria (SF >8 or 3-8 with CRP >45 mg/L) required colectomy. In the same study, the authors reported that patients satisfying these criteria were at high risk for resistance to IV corticosteroids.^10^ In our study, the Oxford criteria was the only factor that was statistically significant in predicting non-response to IV corticosteroids on multivariate analysis indicating the relevance of these simple criteria in the post biologic era.

Other scoring indices that were developed in the prebiologic era (including the SEO index, Lindgren index, and Ho index) were not fully validated and are not commonly used. More recently developed indices are based on more objective parameters such as the CRP/Albumin ratio, ACE index (Albumin, CRP, and Endoscopic severity), as well as a combination of endoscopic severity and biomarkers such as fecal calprotectin (FCAL) have also shown to be predictive of steroid non-response. A recent scoring index developed in the UK and validated in 2 independent cohorts (ADMIT-ASC) was designed as a prediction tool on the day of admission.^16^ Even though these new indices have high specificity and positive predictive value in identifying patients who might not respond to IV corticosteroids, poor sensitivity limits their use. For example, a score of 4 on ADMIT-ASC had a specificity of 100% but sensitivity of only 13%. However, if patients satisfy these criteria on Day 1, rescue therapy could be considered upfront without a trial of corticosteroids. A further limitation of ADMIT-ASC is that the UCEIS is not routinely used for scoring UC disease activity in the majority of centers. Despite the availability of several predictive indices, there is no single accurate predictive index that can determine which patient needs rescue therapy.

Acute severe ulcerative colitis (ASUC) is a dynamic inflammatory condition that requires continuous monitoring and timely intervention. The first 3 days of hospitalization are critical for identifying patients who may need rescue therapy. Early trends in clinical symptoms and biomarkers, such as CRP, can help detect non-response to IV corticosteroids during initial management. Although the Oxford criteria remained significant in multivariate analysis for predicting non-response to IV corticosteroids, changes in CRP and SF from Day 1 to Day 3 were significant predictors in univariate analysis and may serve as useful adjunctive tools in decision making.

## 5. Conclusion

We acknowledge several limitations of our study. First, our study is a retrospective observational study, therefore prone to various biases and there were missing data which could have affected the results. Second, the diagnosis of ASUC was determined by the treating physician based on clinical judgement but not based on TW criteria. Therefore, approximately 18% of the patients did not satisfy the definition of ASUC based on TW criteria because of several reasons including absence of blood in stools, SF <6 despite the presence of systemic inflammatory features. However, our cohort represents real-world practice. Third, we could evaluate only a limited number of scoring indices as endoscopic scores such as UCEIS and biomarkers such as FCAL were not available. Despite these limitations our study offers valuable outcome data in the post biologic era. Last, thromboprophylaxis was administered for only 67% which could be due to missed data. Patients presenting with ASUC are at increased risk of venous thromboembolism and therefore, all patients should be considered for thromboprophylaxis unless contraindicated.

To conclude, although the management of ASUC has improved over the years, and despite a high initial response to corticosteroids, we observed that long-term colectomy rates and re-hospitalization rates are still high, and the risk persists even after successful medical rescue therapy. The simple Oxford criteria was able to predict steroid non-response on Day 3 and can be used to guide management decisions and early intervention with medical or surgical rescue therapy.

## Data Availability

The data that support the findings of this study are available on request from the corresponding author.
